# Enhanced Axonal Extension of Subcortical Projection Neurons Isolated from Murine Embryonic Cortex using Neuropilin-1

**DOI:** 10.3389/fncel.2017.00123

**Published:** 2017-05-01

**Authors:** Noritaka Sano, Takafumi Shimogawa, Hideya Sakaguchi, Yoshihiko Ioroi, Yoshifumi Miyawaki, Asuka Morizane, Susumu Miyamoto, Jun Takahashi

**Affiliations:** ^1^Department of Clinical Application, Center for iPS Cell Research and Application, Kyoto UniversityKyoto, Japan; ^2^Department of Neurosurgery, Kyoto University School of MedicineKyoto, Japan; ^3^Department of Neurosurgery, Graduate School of Medical sciences, Kyushu UniversityFukuoka, Japan; ^4^Department of Neurosurgery, National Hospital Organization Himeji Medical CenterHyogo, Japan

**Keywords:** neuropilin-1, subcortical projection neuron, transplantation, corticospinal tract, cell sorting

## Abstract

The cerebral cortical tissue of murine embryo and pluripotent stem cell (PSC)-derived neurons can survive in the brain and extend axons to the spinal cord. For efficient cell integration to the corticospinal tract (CST) after transplantation, the induction or selection of cortical motor neurons is important. However, precise information about the appropriate cell population remains unclear. To address this issue, we isolated cells expressing Neuropilin-1 (NRP1), a major axon guidance molecule receptor during the early developmental stage, from E14.5 mouse embryonic frontal cortex by fluorescence-activated cell sorting. Aggregates of NRP1^+^ cells gradually expressed subcortical projection neuron markers, Ctip2 and VGluT1, and axon guidance molecule receptors, Robo1 and deleted in colorectal calcinoma (Dcc), *in vitro*, suggesting that they contained early-stage subcortical projection neurons. We transplanted NRP1^+^ cells into the frontal cortex of P2 neonatal mice. Compared with grafts derived from NRP1^−^ or unsorted cells, those derived from NRP1^+^ cells extended a larger number of axons to the spinal cord along the CST. Our data suggest that sorting NRP1^+^ cells from the embryonic cerebral cortex enriches subcortical projection neurons to reconstruct the CST.

## Introduction

Cell-based therapy is a promising treatment for neurodegenerative diseases such as Parkinson’s disease (Lindvall and Hagell, 2000; Doi et al., 2014) and Huntington’s disease (Gallina et al., 2010). Similarly, reconstruction of the corticospinal tract (CST) by cell transplantation is expected as a treatment for stroke and brain injuries. The cortical tissue of murine embryonic brain elongates axons to the spinal cord in neonatal and adult mice (Ebrahimi-Gaillard and Roger, 1996; Gaillard et al., 2007). Neural progenitor cells (NPCs) derived from pluripotent stem cells (PSCs) can extend axons from the cortex to the spinal cord along the CST in neonatal mice (Ideguchi et al., 2010). In rodent stroke models, PSC-derived neurons survive and improve motor dysfunction (Oki et al., 2012; Shinoyama et al., 2013; Tornero et al., 2013). These findings suggest that embryonic cortical tissue and PSC-derived NPCs have the potential to reconstruct the CST, but the cell population that extends axons to the spinal cord upon transplantation remains unknown. To address this issue, we sought a novel cell surface marker for this cell population in the murine embryonic cortex.

Neuropilin-1 (NRP1) was originally found in the optic tectum of Xenopus tadpole and is an axonal guidance molecule receptor essential for the earliest stage of axonal sprouting (Takagi et al., 1987, 1995). At the molecular level, it is a single-pass transmembrane receptor that binds to both semaphorins and vascular endothelial growth factors (VEGF; Raimondi and Ruhrberg, 2013). In the developing cerebral cortex, NRP1 is highly expressed by migrating excitatory neurons and is involved in their migration along radial glial fibers (Chen et al., 2008; Hatanaka et al., 2009). Importantly, NRP1 plays a vital role in initial axonal extension toward the CST (Bagnard et al., 1998).

Cell sorting is a powerful technology that purifies a specific cell population using antibodies. Using this technique, we selected NRP1^+^ cells in the mouse embryonic cortex. When grafted into the frontal cortex of neonatal mice, the sorted NRP1^+^ cells extended axons along the CST more efficiently than unsorted or NRP1^−^ cells. These results suggest that the sorting of NRP1^+^ cells enriches neurons that can reconstruct the CST.

## Materials and Methods

### Cortical Cell Harvesting and Cell Sorting

The frontal cortices of E14.5 mice of either sex (C57BL/6-Tg[CAG-EGFP], RRID:IMSR_RBRC00267) were used for transplantation, and C57BL/6NCrSlc of either sex were used as the hosts. C57BL/6NCrSlc brains were analyzed by immunofluorescence and quantitative real time polymerase chain reaction (RT-PCR). All mice were purchased from Japan SLC (Shizuoka, Japan). The cortices were harvested, gently dissociated into single cell suspensions by Accumax (Innovative cell Technologies, San Diego, CA, USA) and resuspended in phenol-free, Ca^2+^Mg^2+^-free Hank’s balanced salt solution (HBSS; Invitrogen, Waltham, MA, USA) containing 2% FBS, 10 mM Y-27632 (Wako, Osaka, Japan), 20 mM D-glucose (Wako), and 50 mg/ml penicillin/streptomycin (Invitrogen). Samples were filtered through cell-strainer caps (35 μm mesh; BD Biosciences, Franklin Lakes, NJ, USA) and then subjected to surface marker staining using an anti-NRP1 antibody (5.0 × 10^7^ cells/10 μg/1 ml; R and D Systems, Cat# AF566 RRID:AB_355445) as a primary antibody and Alexa 647-conjugated anti-goat IgG (1:400; Invitrogen) as a secondary antibody. The antibodies were added and incubated at 4°C for 30 min, and the cells were washed twice with HBSS buffer. The analysis was performed using a FACSAria II or FACSAria III cell sorter and FACSDiva software (BD Biosciences). A 100 mm ceramic nozzle (BD Biosciences) with a sheath pressure of 20–25 psi and an acquisition rate of 2000–5000 events/s was used for the sorting. Dead cells and debris were excluded by 7-AAD staining. A positive staining gate was set so that less than 0.1% of events exceeded the threshold in samples lacking primary antibodies, and a negative staining gate was set so that less than 1.0% of NRP1^+^ cells were included in the analysis of NRP1^−^ sorted cells. The percentage of NRP1^+^ and NRP1^−^ sorted cells was 24.8 ± 0.8% and 43.7 ± 2.8% of all live single cells, respectively; around 30% of live cells had an intermediate expression of NRP1 and were excluded from further analysis. The sorted cells were collected and replated in U-shaped 96-well low cell adhesion plates (Primesurface, Sumitomo bakelite, Tokyo, Japan; 20,000 cells/well) with culture medium containing Dulbecco’s modified Eagle’s medium (DMEM)/F-12 (Sigma-Aldrich, St. Louis, MO, USA) supplemented with 0.1 mM 2-mercaptoethanol, B27 supplement (without vitamin A, Invitrogen), N2 supplement (ThermoFisher, Waltham, MA, USA), and 25 μM rmFGF-8b (R&D systems, Minneapolis, MN, USA). Half of the medium was changed on day 2, and samples were collected on days 1, 2 and 4.

### Cell Transplantation

Cell aggregates were preserved in culture medium in the 96-well culture plate described above at 37°C for 8–12 h after sorting, transferred to HBSS at 40,000 cells/μl and kept at 4°C until transplantation. Then the aggregates were transplanted into the frontal cortex of neonatal C57BL/6NCrSlc mice aged postnatal day 2 (P2). Briefly, neonatal mice were cryoanesthetized in ice water until they stopped breathing. Then the mice were immobilized on an ice bed and covered by wet gauze, keeping their head at the horizontal position. After checking the absence of pain reflex by pinching the cranial skin with jeweler’s tweezers, a small midline incision in the skin and a small window of the skull over the motor cortex (1.0 mm lateral, 0.5–1 mm anterior from the bregma) was made using fine microscissors and jeweler tweezers. Spheres were then transplanted with a sterile 32 gauge microsyringe (Ito corporation, Shizuoka, Japan) into four sites (0.25 μl/site) targeting the left motor cortex (from the bregma: (1), (2) anterior 0.5 mm, lateral 1.0 mm, vertical 0.4 and 0.7 mm, (3), (4) anterior 1 mm, lateral 1.0 mm, vertical 0.4 and 0.7 mm) over 1 min. The skin incision was closed with surgical 10–0 sutures (BEAR Medic, Ibaraki, Japan), and the pups were resuscitated on a warming pad. To reduce the negative influence of cryoanesthesia, the duration of the hypothermia was kept under 10 min. Thirty one days after transplantation, mice were anesthetized with 3% isoflurane and clamped in a stereotactic apparatus (Narishige, Tokyo, Japan). Following linear incision of the skin overlying a cervical region, laminectomy of C1 was performed, and 0.3 μl of 4% Fast blue (Polysciences, Warrington, PA, USA) and 4% Dimethyl sulfoxide (Sigma-Aldrich, St. Louis, MO, USA) in artificial cerebrospinal fluid (Harvard Apparatus, Holliston, MA, USA) was injected into the posterior column at C1–2 level. Thirty-four days after transplantation, mice were transcardially perfusion-fixed with 4% paraformaldehyde (PFA; Wako), and the brains and spinal cords were fixed with 4% PFA for 6 h, transferred to 30% sucrose (Nacarai Tesque, Kyoto, Japan) in PBS, and preserved at 4°C. They were then embedded with O.C.T. compound (Sakura finetek, Torrence, CA, USA), cut with a cryostat (Leica Biosystems, Buffalo Grove, IL, USA) into 50-μm sections and preserved in antifreeze (30% glyceol [Nacalai tesque], 30% ethylene glycol [Wako] and 40% PBS) at −30°C before use. The number of ipsilateral-projecting axons derived from a graft was counted in the coronal section at: (1) the cerebral peduncle, (2) the pyramidal tract in the pons, and in the longitudinal section of (3) the spinal posterior column lower than C3 level. In the coronal sections, two sections from the site of interest in each animal were labeled by anti-GFP antibody, and the mean numbers of neurites were recorded. In the longitudinal sections, the largest number of neurites in each case was recorded. In each group, 15 animals were initially enrolled, but three animals in each group were excluded due to poor graft survival or improper engraftment into the internal capsule or the contralateral cortex. The remaining 12 animals in each group were used in the study.

Animals were cared for and handled according to the Guidelines for Animal Experiments of Kyoto University and the Guide for the Care and Use of Laboratory Animals of the Institute of Laboratory Animal Resources (ILAR; Washington, DC, USA).

### Quantitative Real Time PCR

Total RNA was isolated using an RNeasy Plus Mini Kit (QIAGEN, Venlo, Netherlands), and cDNA was synthesized from more than 50 ng of RNA using a SuperScript III First-Strand Synthesis System (Invitrogen). Quantitative PCRs were carried out with SYBR Premix Ex Taq (TaKaRa, Kusatsu, Japan) and the Thermal Cycler Dice Real Time System (TaKaRa). The data were assessed using the ΔΔ-Ct method and normalized by the GAPDH expression. The primer sequences used are shown in Table 1.

**Table 1:** **Primers used for polymerase chain reaction (PCR)**.

Gene	Forward (5′–3′)	Reverse (3′–5′)
*GAPDH*	CCGCCTGGAGAAACCTGCCAAGT	GGGAGTTGCTGTTGAAGTCGCAGG
*VGluT1*	GCCTTTTGCGGTTCCTATGC	AAAGATCCCGAAGCTGCCAT
*VGluT2*	AACAAAGGATTTTGGCCCCG	CAGCACCCTGTAGATCTGTCC
*Ctip2*	ACCCACGAAAGGCATCTGTC	GGAACCAGGCGCTTGTTGAA
*Fezf2*	GGAGGGGAAGATGTTTGCCA	TCCTCTAAGTCTCTTTCCCCCA
*Unc5D*	ACCCCGCTATACCCTCT	TGCCTTCCCGGCTTTAT
*NeuroD1*	CTGTCAGAGATCCTG	GCTGGGACAAACCTT
*Tbr2*	TGTGACGGCCTACCAAAACA	AGCCGTGTACATGGAATCGT
*Pax6*	CGTAGAACCCGGTTGTCAGA	AAGTCTTCTGCCTGTGAGCC
*Robo1*	GCTGGCGACATGGGATCATA	TTACAACGAAATGTGGCGGC
*Dcc*	AACAATGCCGGAGAAGGTGT	CGGGGTCAGTGGGATCTGTT

### Immunostaining

On days 2 (36 h) and 4 (84 h) from the day of sorting, aggregates were fixed in 4% PFA and washed with and preserved in PBS at 4°C until use. The aggregates were embedded with 20% O.C.T. compound (Sakura finetek) in PBS, cryosectioned into 14-μm sections using Cryostat (Leica), and attached to an MAS-coated glass slide (Matsunami, Osaka, Japan). Double immunohistochemical analysis of the cryosections (aggregates, brains and spines) were carried out after permeabilization with 2% Triton X-100 and blocking in 4% Block ACE (DS pharma biomedical, Osaka, Japan). The immunoreactive cells were visualized using a fluorescence microscope (BZ-9000; Keyence, Osaka, Japan) or confocal laser microscope (Fluoview FV1000; Olympus, Tokyo, Japan). The primary antibodies used were as follows: anti-NRP1 (1:250, ECM Biosciences Cat# NP2111 RRID:AB_2155222), Anti-Tbr1 (1:500, Abcam Cat#ab31940 RRID: AB_2200219), Anti-Ctip2[25B6] ChIP Grade (1:500, Abcam Cat# ab18465 RRID:AB_2064130), Anti-Satb2 (1:200, Abcam Cat# ab51502 RRID:AB 882455), Anti-NeuN (1:500, Millipore Cat# MAB377 RRID:AB2298772), anti-NeuroD (1:200, Santa Cruz Cat# sc1084 RRID:AB_630922), anti-Tbr2 (1:500, Abcam Cat# ab23345 RRID:AB_778267), anti-Ki67(1:1000, Novocastra, Cat# KCL-ki67p), anti-Pax6(1:500, Covance, Cat# PRB-278P RRID:AB_2565003) anti-Nestin (1:1000, Millipore, Cat# MAB353 RRID:AB_94911), anti-VGluT1/2 (1:2000, Synaptic Systems Cat# 135 303 RRID:AB_887876), anti-GAD2 (1:500, Millipore Cat# MAB351 RRID:AB_2263126), GSL I-Isolectin B4 (IB4; 1:50, Vector laboratories Cat# DL-1207 RRID:AB_2336415) anti-Doublecortin (DCX; 1:1000, Santa Cruz Biotechnology Cat# sc-8066 RRID:AB_2088494), anti-GFP (1:1000, MBL International, Cat# 598 RRID:AB_591819), and anti-myelin basic protein (MBP; 1:1000, Millipore, Cat# MAB386 RRID:AB_94975).

### Statistical Analysis

The statistical analyses were performed using JMP 11 (SAS Institute, Cary, NC, USA). For the comparison of two groups, the significance of differences was determined by one-way analysis of variance (ANOVA), and of three groups, one-way ANOVA followed by Tukey’s *post hoc* test was used. The differences were considered statistically significant when probability values were less than 0.05. The data are presented as the mean ± standard error of the mean (SEM).

## Results

### Migrating and Projecting Neurons were Enriched by Sorting NRP1^+^ Cells

**Figure 1:**
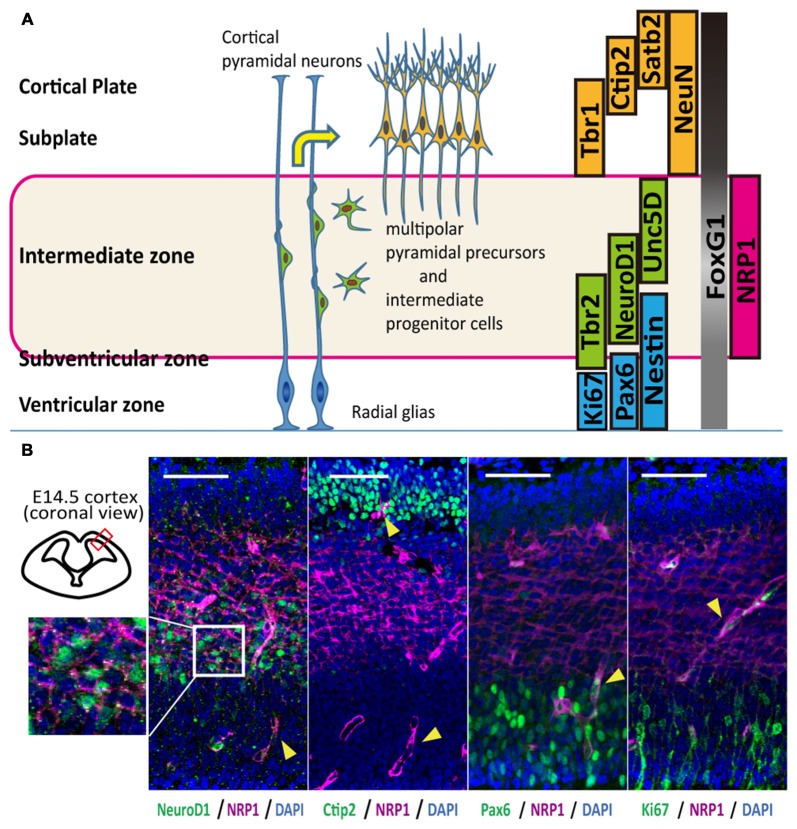
**Anatomical distribution and function of Neuropilin-1 (NRP1) as an axonal guidance molecule in different developmental stages. (A)** Schematic diagram of the maturation of cortical pyramidal neurons and markers related to the cortical layers. **(B)** Immunohistological analysis of the anatomical NRP1 (magenta) distribution in E14.5 mouse cortex. Arrowheads indicate blood vessels. Scale bars, 50 μm.

In mice, corticospinal motor neurons (CSMNs) initiate axonal extension at embryonic day 13–14 (E13–14; Canty and Murphy, 2008), and the frontal cortex at this age contains neurons that construct the CST (Ebrahimi-Gaillard and Roger, 1996; Gaillard et al., 2007). The cerebral cortex of E14 is divided into four layers that include the cortical plate and the intermediate (IZ), subventricular (SVZ) and ventricular (VZ) zones, and each layer is characterized by specific markers (Figure 1A). Most of the neurons in the cortical plate and subplate are glutamatergic-expressing VGluT1 (El Mestikawy et al., 2011), and migrating neurons in the IZ and SVZ during the duration of interest express VGluT2 (Ina et al., 2007). Subcortical projection neurons, collosal projection neurons and postmitotic neurons from the subplate to layer VI express Ctip2, Satb2 and Tbr1, respectively. Some overlap of the expression exists, however, and all these cortical plate neurons express NeuN. The IZ is characterized by markers for multipolar pyramidal neurons such as NeuroD1 and Unc5D (Miyoshi and Fishell, 2012). Intermediate progenitor cells in the SVZ are positive for Tbr2, while progenitors in the VZ express Pax6 (Englund et al., 2005). In the frontal cortex, all cells express a telencephalic marker, FoxG1, especially those in the cortical plate (Figure 1A).

**Figure 2:**
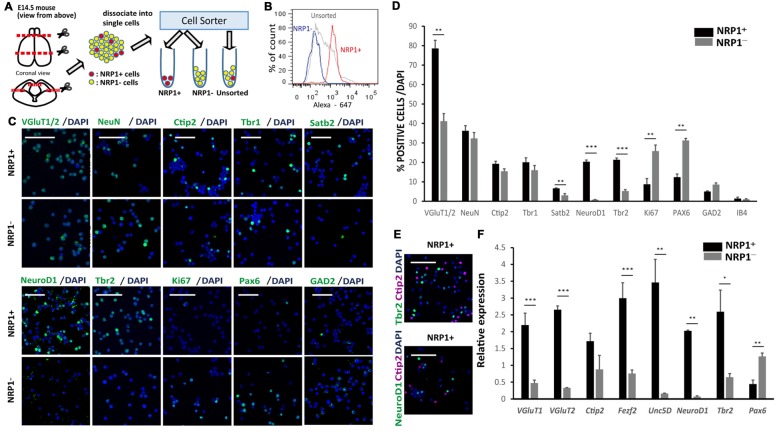
**Characterization of murine cerebral cortex-derived NRP1^+^ cells immediately after cell sorting. (A)** The cell sorting procedure. Rostral 2/3 of E14.5 cerebral cortex is harvested, dissociated by Accumax^®^ into single cells, and divided into three groups (NRP1^+^, NRP1^−^ and Unsorted). **(B)** A histogram of the fluorescence-activated cell sorting (FACS) analysis of NRP1^+^, NRP1^−^ and unsorted cells. **(C)** Immunostaining of NRP1^+^ and NRP1^−^ cells for VGluT1/2, NeuN, Ctip2, Tbr1, Satb2, NeuroD1, Tbr2, Ki67, Pax6 and GAD2 (green)/DAPI (blue). Scale bars, 50 μm. **(D)** Frequency distribution of several neural markers as a percentage of total DAPI stained cells in each group. **(E)** Immunostaining of NRP1^+^ cells for Tbr2 (green)/Ctip2 (magenta)/DAPI (blue) and NeuroD1 (green)/Ctip2 (magenta)/DAPI (blue). Scale bars, 50 μm. **(F)** Quantitative real time polymerase chain reaction (RT-PCR). The expression level of unsorted cells was set to 1. Values are the mean ± SEM. *p < 0.05, **p < 0.01 and ***p < 0.001 by one way analysis of variance (ANOVA; *n* = 3 independent experiments).

An immunofluorescence study of the E14.5 mouse cortex revealed that NRP1 is expressed on the cell bodies and neurites in the IZ and outer SVZ, and all NeuroD1^+^ cells co-expressed NRP1. In contrast, postmitotic pyramidal neuron markers such as Ctip2 or NeuN were rarely colocalized with NRP1, and NRP1^+^PAX6^+^ cells observed only in the SVZ (Figure 1B). Therefore, it is assumed that one of the NRP1^+^ cell populations are the subcortical projection neurons in the cortical plate, which express NRP1 only in the axons in the IZ and SVZ. Another one is migrating excitatory neurons in the IZ and SVZ, which express NRP1 in both the cell bodies and axons. To confirm this assumption, we sorted NRP1^+^ cells from the frontal cortex of E14.5 mice by fluorescence-activated cell sorting (FACS; Figure 2A). The percentages of NRP1^+^ and NRP1^−^ cells were 24.8 ± 0.8% and 43.7 ± 2.8%, respectively (Figure 2B). The remaining cells showed intermediate expression of NRP1 and thus were excluded from the following analyses.

An immunofluorescence study of sorted cells revealed that 78.6 ± 4.2% of NRP1^+^ cells expressed VGluT1/2, suggesting that they are excitatory neurons in the cortical plate IZ, and SVZ (Figures 2C,D). NRP1^+^ neurons in the cortical plate that also expressed Ctip2, Tbr1 and Satb2 were 19.2 ± 1.4, 20.1 ± 2.3 and 6.7 ± 0.2%, respectively, suggesting that they were projection neurons with axonal extensions. In addition, 20.3 ± 0.9 and 21.3 ± 0.9% of the NRP1^+^ cells expressed NeuroD1 and Tbr2, respectively, suggesting that they were migrating excitatory neurons in the IZ and SVZ. The expression of Ctip2 never overlapped with the expression of NeuroD1 or Tbr2 (Figure 2E). Considering that NeuN^+^ cortical neurons were 36.3 ± 2.6% of the NRP1^+^ population, excitatory NRP1^+^ neurons in the IZ or SVZ accounted for the other 42%. For minor populations, 12.4 ± 1.6% of NRP1^+^ cells expressed Pax6 (Figures 2C,D). Because NRP1 is not expressed in the VZ, NRP1^+^Pax6^+^ cells were assumed to be newly developed migrating neurons in the SVZ or radial glia without a process contacting the ventricular surface. NRP1^+^ cells also included GABAergic neurons expressing GAD2 (5.0 ± 0.3%) and vascular endothelial cells expressing IB4 (1.4 ± 0.7%).

In contrast, the major population of NRP1^−^ cells was Pax6^+^ cells (31.3 ± 1.1%). Moreover, most of the PAX6^+^ cells expressed a marker for proliferating cells, Ki67, suggesting that they were NPCs in the VZ. 32.3 ± 3.1% of NRP1^−^ cells were NeuN^+^ cortical neurons. NRP1^−^ cortical neurons that expressed Ctip2, Tbr1 and Satb2 were 15.5 ± 1.4, 16.0 ± 2.4 and 3.0 ± 0.9%, respectively. NRP1^−^ cells also contained a small percentage of GAD2^+^ cells (8.6 ± 0.8%) and IB4^+^ cells (1.0 ± 0.4%). Thus, the major population of NRP1^+^ cells was migrating excitatory neurons in the IZ and SVZ, and that of NRP1^−^ cells was NPCs in the VZ. These observations were confirmed by a quantitative PCR analysis, which showed that NRP1^+^ cells expressed higher levels of several IZ and SVZ markers, including *Tbr2*, *NeuroD1* and *Unc5D*, compared with unsorted cells (Figure 2F). It is also noteworthy that NRP1^+^ cells expressed higher levels of CMSN markers such as *VGluT1*, *Ctip2* and *Fezf2*. In contrast, NRP1^−^ cells expressed higher levels of *Pax6*, and the expression levels of IZ markers were very low.

### NRP1^+^ Cells Differentiated into Subcortical Projection Neurons *In Vitro*

**Figure 3:**
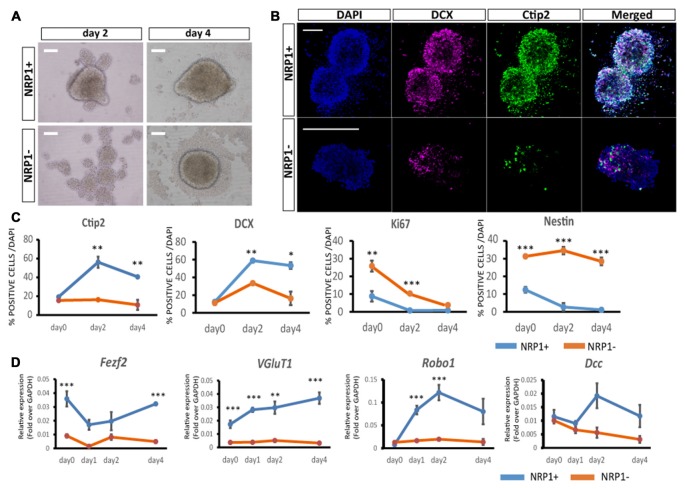
**Characterization of aggregates by immunostaining. (A)** Bright-Field images of NRP1^+^ and NRP1^−^ aggregates on days 2 and 4. Scale bars, 100 μm. **(B)** Immunostaining of day 2 NRP1^+^ and NRP1^−^ aggregates. Doublecortin (DCX; magenta)/Ctip2 (green)/DAPI (blue). Scale bars, 100 μm. **(C,D)** Time course analysis of immunostaining for **(C)** Ctip2, DCX, Ki67 and Nestin positive cells as a percentage of total DAPI stained cells on days 0, 2 and 4. **(D)** Time course qPCR analysis of days 0, 1, 2 and 4 aggregates. Values are the mean ± SEM. *p < 0.05, **p < 0.01 and ***p < 0.001 by one way ANOVA (*n* = 3 independent experiments).

To elucidate the differentiation propensity of NRP1^+^ cells, we cultured the sorted cells in DMEM/F-12/N2/B27-based medium in 96 well low-attach plates (20,000 cells/well). NRP1^+^ cells formed aggregates in 12 h, whereas NRP1^−^cells took 2–3 days (Figure 3A). An immunofluorescence study revealed that the percentages of cells expressing Ctip2 and DCX, a marker for CSMNs and neurons with the capacity of extending neurites, respectively, were increased during 4 days of culture only in NRP1^+^ cells (Figures 3B,C). In contrast, Ki67^+^ and Nestin^+^ cells almost disappeared in the NRP1^+^ cell population (Figure 3C). Quantitative PCR analysis disclosed that *Fezf2* and *VGluT1* expressions was higher in NRP1^+^ cells (Figure 3D). With regards to the expression of receptors that act as axonal guidance cues for CSMNs, *Robo1* and *deleted in colorectal calcinoma* (*Dcc*) peaked on day 2 only in NRP1^+^ aggregates, but were relatively unchanged in NRP1^−^ cells (Figure 3D). These results indicated that NRP1^+^ cells have the ability to differentiate into subcortical projection neurons.

### NRP1^+^ Cells Survived in the Forebrain and Extended Axons along CST

**Figure 4:**
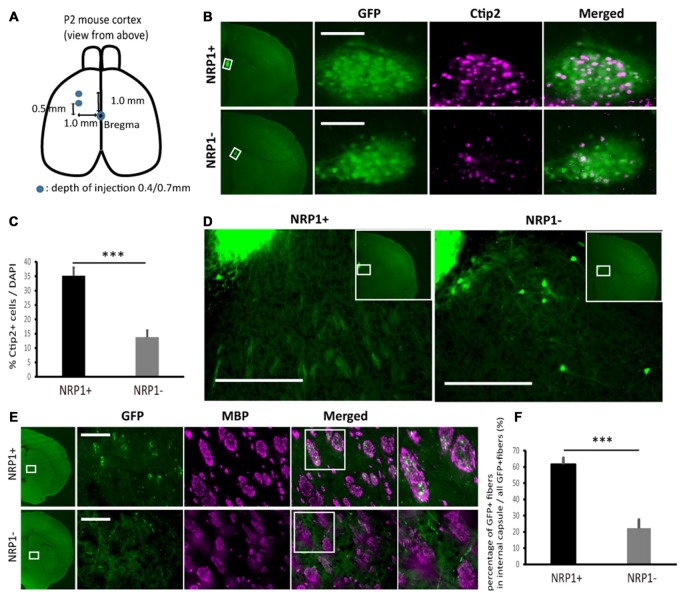
**Characterization of sorted cell-derived grafts and graft-derived neurites in fasciculated bundles in medial striatum. (A)** Schematic of the transplantation. Cryoanesthetized pups were fixed on a bed, and cells (4.0 × 10^4^ cells in total) were injected to the points (blue) depicted in the figure. **(B)** Immunostaining of representative grafts for GFP (green)/Ctip2 (magenta). Scale bars, 100 μm. **(C)** Frequency of Ctip2 as a percentage of total DAPI stained cells in the graft. Values are the mean ± SEM. *F*_(1,22)_ = 33.2, *p* < 0.0001 and ***p < 0.001 by one way ANOVA. (NRP1^+^, *n* = 12; NRP1^−^, *n* = 12). **(D)** Immunostaining of representative extension patterns of neurites around the grafts using the same section as that in Panel **(B)**. Scale bars, 100 μm. **(E)** Representative images of GFP^+^ neurites (green) in myelin positive fasciculated bundles (magenta) in the medial striatum. Scale bars, 100 μm. **(F)** Frequency of GFP^+^ fibers in the fasciculated bundles in the medial striatum (myelin basic protein (MBP) positive area, magenta) as a percentage of all GFP^+^ cells in the same region. Values are the mean ± SEM. *F*_(1,22)_ = 35.4, *P* < 0.0001. ***p < 0.001 by one way ANOVA. (NRP1^+^, *n* = 12; NRP1^−^, *n* = 12).

Next, to investigate the survival and axonal extension *in vivo*, we grafted NRP1^+^, NRP1^−^ and unsorted cells into the forebrain of neonatal mice (Figure 4A). We isolated donor cells from GFP knock-in mice (Okabe et al., 1997) to distinguish them from host cells. The grafts were mainly distributed in deep cortical layer adjacent to the corpus callosum (CC) in the frontal cortex. Five weeks after transplantation, the number of surviving GFP^+^ cells in the NRP1^+^ and NRP1^−^ cell-derived grafts were 2.4 ± 0.5 × 10^3^ and 1.8 ± 0.5 × 10^3^, respectively. The NRP1^+^ cell-derived grafts contained more Ctip2^+^ cells than did NRP^−^cell-derived grafts (35.2 ± 2.8% vs. 13.8 ± 2.4%; Figures 4B,C). NRP1^+^ graft extended numerous neurites around the graft and formed bundles in the striatum, while NRP1^−^ graft extended fewer neurites and some grafted cells migrated into the striatum (Figure 4D). CSMN axons are known to make highly fasciculated and tight bundles in the medial striatum (Arlotta et al., 2005), and these bundles are surrounded by MBP (Lodato et al., 2014). Immunostaining using anti-MBP antibody revealed that NRP1^+^ cells extended fasciculated axons in the MBP^+^ area more frequently than NRP^−^ cells (61.6 ± 4.0 vs. 22.2 ± 5.3%; Figures 4E,F). These results supported the idea that NRP1^+^ cells survive in the forebrain and extended axons along the CST.

### NRP1^+^ Cells Efficiently Extend Axons along the CST to the Spinal Cord

**Figure 5:**
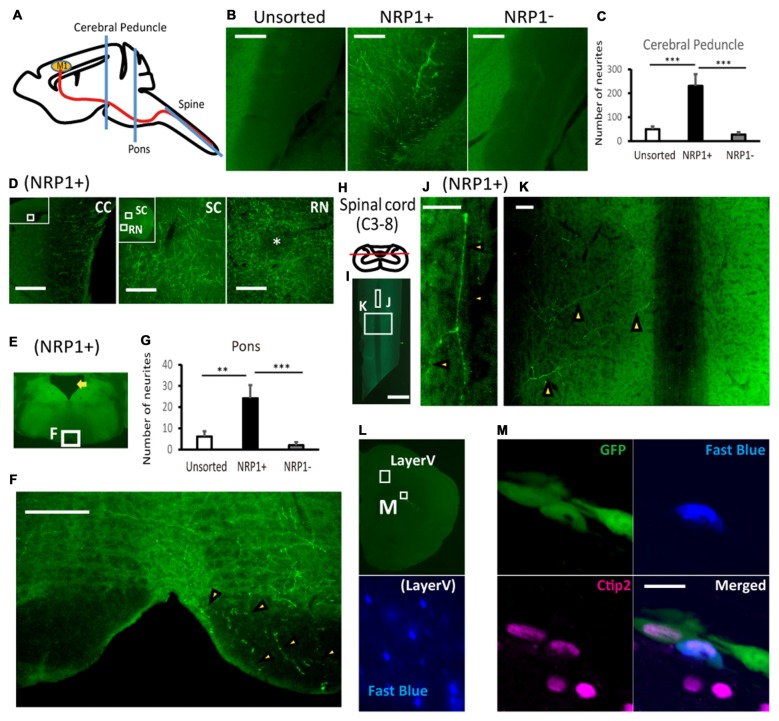
**NRP^+^ cell-derived grafts project more neurites along the corticospinal tract (CST) to the spinal cord. (A)** Schematic diagram of the section line at the cerebral peduncle, pons and spinal cord. M1, primary motor cortex. **(B)** Representative images of GFP^+^ neurites (green) at the cerebral peduncle in NRP1^+^, NRP1^−^ and unsorted cells. Scale bars, 100 μm. (unsorted, *n* = 12; NRP1^+^, *n* = 12; NRP1^−^, *n* = 12). **(C)** Enumeration of GFP positive neurites at the internal capsule. Values are the mean ± SEM. *F*_(2,33)_ = 13.7, *p* < 0.0001. ***p < 0.001 by one way ANOVA followed by Tukey’s *post hoc* test. **(D)** Representative images of GFP^+^ neurites in the contralateral corpus callosum (CC), around the ipsilateral superior colliculus (SC) and red nucleus (RN) in the NRP1^+^ group. Scale bars, 100 μm. **(E)** Low power image of the pons. The arrow indicates the 4th ventricle. **(F)** Representative figures of GFP^+^ neurites (arrowheads) at the lower pons in the NRP1^+^ group. Scale bar, 100 μm. **(G)** Enumeration of GFP^+^ neurites at the pons. Values are the mean ± SEM. *F*_(2,33)_ = 9.3, *p* = 0.0006. **p < 0.01 and ***p < 0.001 by one way ANOVA followed by Tukey’s *post hoc* test. (unsorted, *n* = 12; NRP1^+^, *n* = 12; NRP1^−^, *n* = 12). **(H)** Schematic diagram of the section line parallel to the pyramidal tract in the posterior column. **(I)** Low power image of the cutting plane including the posterior column of the spinal cord (C3-Th2). Scale bar, 1000 μm. **(J,K)** High power image of **(J)** the posterior column and **(K)** right spinal layer. Arrowheads indicate GFP^+^ neurites, and there was no neurites in the left spinal layer. Scale bars, 50 μm. (unsorted, *n* = 12; NRP1^+^, *n* = 12; NRP1^−^, *n* = 12). **(L)** Low power image of left frontal cortex. An appropriate injection of fast blue was confirmed at layer V neurons as positive control. **(M)** High power image of the graft in **(L)**. Immunostaining for GFP (green)/Ctip2 (magenta)/Fast blue (blue). Scale bars, 25 μm.

Finally, we evaluated the axonal extension of the grafted cells by counting the number of GFP^+^ neuronal fibers at the cerebral peduncle, pons and spinal cord (Figure 5A). At the ipsilateral cerebral peduncle, the number of axons was significantly higher in the NRP1^+^ cell-derived grafts than either NRP^−^ or unsorted grafts (230.4 ± 49.7, 27.5 ± 9.8 and 49.8 ± 11.9, respectively; Figures 5B,C). In addition, in NRP1^+^ cell-derived grafts, neurites were found in the contralateral CC, the ipsilateral superior colliculus (SC) and red nucleus (RN), which are other physiological targets of callosal projection neurons and subcortical projection neurons (Figure 5D). At the ipsilateral pons (Figure 5E), the number of axons was significantly higher in the NRP1^+^ cell-derived grafts than either NRP^−^ or unsorted grafts (24.2 ± 6.2, 2.0 ± 1.5 and 6.2 ± 2.4, respectively), and no axons were found in the contralateral side of the grafts (Figures 5F,G). At the spinal cord (Figure 5H), GFP^+^ axons were observed only in the NRP1^+^ group (2.6 ± 0.8), and all GFP^+^ axons seemed to innervate gray matter contralateral to the graft site (Figures 5I–K). To confirm that the axons were derived from the grafted cells, we injected Fast blue, a retrograde tracer, into the posterior column of C1 level of the spinal cord. Double-labeled staining revealed that all Fast blue^+^/GFP^+^ cells expressed Ctip2, indicating that the grafted embryonic cells differentiated into cortical Ctip2-expressing cells and extended axons to the spinal cord (Figures 5L,M). These results suggested that the grafted NRP1^+^ cells extended axons along the CST to the spinal cord more efficiently than did NRP1^−^ cells.

## Discussion

We show that NRP1^+^ cells derived from mouse embryonic cortex survived and extended axons more efficiently than NRP1^−^ cells. Furthermore, NRP1^+^ cells possessed the ability to integrate into mouse brain and expressed the subcortical projection neuron marker Ctip2. More importantly, the axons ran along fasciculated bundles in the medial striatum and extended to the spinal cord. These data suggest that NRP1 can be used to isolate subcortical projection neurons that reconstruct the CST.

For the efficient extension of axons along the CST, a coordinated regulation of molecular guidance cues that navigate axons from the deep layer of the frontal cortex to the spinal cord is necessary. Among the cell surface antigens that interact with these cues, NRP1 is essential for the earliest stage of axonal sprouting. NRP1 is mainly distributed in the IZ of the entire E13.5–15.5 mouse cortex (Kawakami et al., 1996; Bagnard et al., 1998; Hatanaka et al., 2009), and *NRP1* mRNA is absent in proliferative cells in the VZ (Takagi et al., 1995). NRP1^+^ cells can include: (1) migrating excitatory neurons in the IZ (Hatanaka and Yamauchi, 2013; Hatanaka et al., 2016); (2) subcortical projection neurons in the cortical plate (Bagnard et al., 1998); (3) callosal projection neurons (Hatanaka et al., 2009); (4) migrating GABAergic interneurons (Marin et al., 2001); and (5) vascular endothelial cells (Kawasaki et al., 1999; Fantin et al., 2013). On the other hand, our results indicate NPCs in the VZ were sorted in the NRP1^−^ cell population.

It was reported that migrating excitatory neurons (multipolar pyramidal precursors) extend axons within the IZ before reaching the cortical plate by a static microscopy (Shoukimas and Hinds, 1978), which was confirmed by a recent study using time-lapse images (Hatanaka and Yamauchi, 2013). In addition, almost all these migrating excitatory neurons express NRP1 (Hatanaka et al., 2009), which is consistent with NRP1 being required for the axonal sprouting of excitatory neurons. Migrating excitatory neurons in the IZ also express NeuroD1 and/or Unc5D (Miyoshi and Fishell, 2012). Consistently, the NRP1^+^ cells in our study expressed higher levels of *NeuroD1*, *Unc5d* and *VGluT1* than did NRP1^−^ cells (29 fold, 22 fold and 4.6 fold, respectively). Almost all NeuroD1^+^ cells were sorted in the NRP1^+^ cell population and accounted for 20.3% of NRP1^+^ cells, while in the NRP1^−^ cell population, only 0.8% of cells expressed NeuroD1. For the reasons stated above, almost all migrating excitatory neurons in the IZ were assumed to be NRP1^+^ cells. Some excitatory neurons express NRP1 only in the distal part of extending neurites such as the growth cone and filopodia (Takagi et al., 1995). This feature is true for Ctip2^+^ subcortical projection neurons in the cortical plate, which express NRP1 only in neurites extending through the IZ (Bagnard et al., 1998). In our study, sorted NRP1^+^Ctip2^+^ cells never overlapped with NRP1^+^Tbr2^+^ or NRP1^+^NeuroD1^+^ cells. These results suggest that NRP1^+^ cells also contained subcortical projection neurons with axonal extensions. NRP1 is also related to the development of callosal projection neurons, which express Satb2 in the cortical plate (Hatanaka et al., 2009). Consistently, the percentage of Satb2^+^ cells were significantly higher in the NRP1^+^ cell population than NRP1^−^ cell population (6.7% vs. 3.0%). During E13.5–15.5, GABAergic interneurons tangentially migrate into the cortex from the striatum and express NRP1 during migration in the IZ (Marin et al., 2001). However, these inhibitory neurons account for only a small portion of NRP1^+^ cells in the IZ, whereas the majority are migrating excitatory neurons (Hatanaka et al., 2009). Consistently, we observed a small amount of GAD2^+^ cells in both NRP1^+^ and NRP1^−^ cell populations. According to these results, the major components of NRP1^+^ cells were migrating excitatory neurons in the IZ and subcortical projection neurons in the cortical plate with axonal extensions.

In our transplantation study, NRP1^+^ cell-derived grafts contained more Ctip2^+^ cells than did NRP1^−^ cell-derived grafts and extended more axons that formed fasciculated bundles in the medial striatum as far as the spinal cord. Ctip2 and/or Fezf2 positive cells extend axons as fasciculated bundles in the medial striatum during the embryonic-neonatal stage (Arlotta et al., 2005; Lodato et al., 2014). Therefore, the formation of axonal bundles by NRP1^+^ cells suggest that these cells are suitable for the regeneration of the CST, but the detailed mechanism remains to be explored.

It is also noteworthy that the expression of Ctip2 and DCX increased only in the case of 4-day culture of NRP1^+^ cells. In addition, the expression of *Robo1* and *Dcc* was also increased only in NRP1^+^ cells. These two receptors are related to the axonal guidance of CSMNs to the internal capsule (Bagri et al., 2002) and midline crossing at the pyramidal decussation (Finger et al., 2002), respectively. Therefore, the higher expression of these CSMN-related molecules supports the advantage of NRP1^+^ cells.

When grafted into the neonatal brain, NRP1^+^ cells extended axons to the cerebral peduncle, pons and lower cervical spinal cord. NRP1^+^ axons, however, were also observed outside the CST, such as in the RN of the SC. The axons were guided to these other targets probably because NRP1 plays a key role in the early phase of axonal guidance but not late phase. NRP1^+^ axons were also observed in the contralateral CC. This observation reflects the result that a subpopulation of NRP1^+^ cells expressed Satb2, a marker for callosal projection neurons in the cortical plate (Alcamo et al., 2008; Hatanaka et al., 2009).

In this study, we grafted the fetal cells in the neonatal brain, where the axonal extension is still in progress as normal development. In the adult brain, however, we cannot expect such a mechanism. Previous report suggests that the grafted fetal neurons can recognize molecular guiding cues re-expressed following CST lesion in the adult brain (Gaillard et al., 2007). Therefore, optimization of the host brain environment along the CST by enhancing or adding such supportive factors is another key to the successful cell therapy for cerebral stroke or brain injury.

In conclusion, we show that NRP1^+^ cells in the frontal cortex of E14.5 mice survive and extend axons to the spinal cord of neonatal brain. These results contribute to the identification of progenitor cells for CSMNs. In addition, if combined with the induction of frontal cortex from PSCs, they could contribute to the development of cell-based therapies to treat CST damage by stroke or brain injury.

## Author Contributions

NS, AM, SM and JT designed the research; NS, TS, YI and YM performed the research, NS TS, HS and JT analyzed the data, and NS, HS and JT wrote the article.

## Conflict of Interest Statement

The authors declare that the research was conducted in the absence of any commercial or financial relationships that could be construed as a potential conflict of interest.
